# Systematic substitutions at BLIP position 50 result in changes in binding specificity for class A β-lactamases

**DOI:** 10.1186/s12858-017-0077-1

**Published:** 2017-03-06

**Authors:** Carolyn J. Adamski, Timothy Palzkill

**Affiliations:** 1 Department of Biochemistry and Molecular Biology, Baylor College of Medicine, Houston, TX USA; 2 Department of Pharmacology, Baylor College of Medicine, Houston, TX USA

**Keywords:** β-lactamase, Binding specificity, Systematic substitutions, Beatmusic

## Abstract

**Background:**

The production of β-lactamases by bacteria is the most common mechanism of resistance to the widely prescribed β-lactam antibiotics. β-lactamase inhibitory protein (BLIP) competitively inhibits class A β-lactamases via two binding loops that occlude the active site. It has been shown that BLIP Tyr50 is a specificity determinant in that substitutions at this position result in large differential changes in the relative affinity of BLIP for class A β-lactamases.

**Results:**

In this study, the effect of systematic substitutions at BLIP position 50 on binding to class A β-lactamases was examined to further explore the role of BLIP Tyr50 in modulating specificity. The results indicate the sequence requirements at position 50 are widely different depending on the target β-lactamase. Stringent sequence requirements were observed at Tyr50 for binding *Bacillus anthracis* Bla1 while moderate requirements for binding TEM-1 and relaxed requirements for binding KPC-2 β-lactamase were seen. These findings cannot be easily rationalized based on the β-lactamase residues in direct contact with BLIP Tyr50 since they are identical for Bla1 and KPC-2 suggesting that differences in the BLIP-β-lactamase interface outside the local environment of Tyr50 influence the effect of substitutions.

**Conclusions:**

Results from this study and previous studies suggest that substitutions at BLIP Tyr50 may induce changes at the interface outside its local environment and point to the complexity of predicting the impact of substitutions at a protein-protein interaction interface.

## Background

Interactions between proteins play an essential role in nearly every cellular process. Each protein in a cell is estimated to interact with approximately five other proteins, forming a complex interaction network [1]. A better understanding of protein-protein interactions, in particular what regulates rates of formation and dissociation and the molecular basis of specificity, would have applications ranging across fields from protein engineering to drug design [2]. Numerous protein-protein interactions have been studied and provide details about the roles of shape complementarity, long- and short-range interactions and solvent in binding [3–10]. However, even with this large accumulation of data, prediction programs often have limited success, largely because of challenges posed by cooperativity between residues, flexibility, and rearrangement at the large, multifaceted interface upon binding [5, 11]. Some success has been shown for predicting changes upon mutation to alanine; however, predicting the effects of mutations to the other 19 amino acids often falls short because residues other than alanine lose interactions and also have the ability to form new ones. It is important to understand how mutations to all possible amino acids modify protein-protein interactions for protein engineering and because mutations other than alanine are frequently seen in nature.

Various protein-protein complexes such as BLIP and β-lactamases have developed into model systems to examine the basic principles underlying protein-protein interactions [6, 7, 12–15]. Studies of a variety of protein complexes indicate that only a subset of residues at the interface contributes substantially to binding affinity and these residues are termed “hot spots” [12, 13, 16–19]. Specificity determinants, i.e., residues where energetic contributions vary depending on the binding partners, were found among the hot spot residues in the BLIP-β-lactamase interaction [12]. Specificity determinants are of particular interest because of their ability to exhibit a significantly different effect on binding affinity to different protein binding partners when mutated. For example, when BLIP Tyr50 was mutated to alanine it exhibited a 50-fold increase in binding affinity for one binding partner (TEM-1) and a 65-fold decrease in binding affinity for another (Sme-1) (Table 1) [12].

**Table 1:** Binding Constants of BLIP and BLIPY50A for Class A β-lactamases

BLIP	Ki (nM) TEM-1	Ki (nM) SHV-1	Ki (nM) Sme-1
WT	0.5^a^	1130^a^	2.4^a^
Y50A	0.011^a^	34^a^	32^a^

^a^values from Zhang Z, et al. [13]

**Fig. 1:**
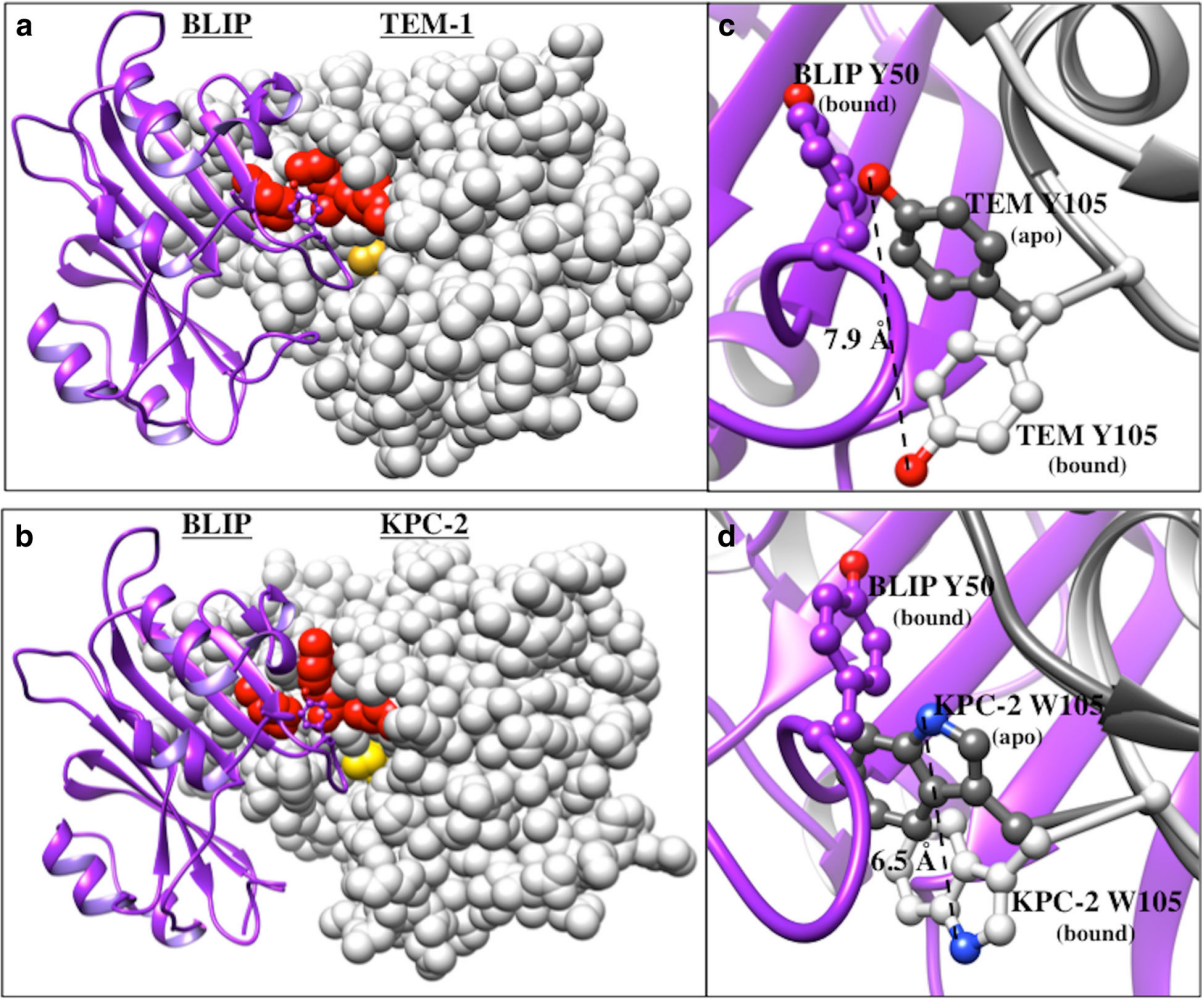
Structural representation of the interaction between BLIP and β-lactamases. BLIP is shown as a purple ribbon with Tyr50_BLIP_ shown as stick. TEM-1 **a** and KPC-2 **b** β-lactamases are shown as *white* spheres with the catalytic Ser70 in *yellow* and positions 107, 129 and 216 (that make contact with Tyr50_BLIP_) are shown in *red*. PDB codes: 1JTG and 3E2K. Alignment of apo (*gray*) and bound (*white*) TEM-1 **c** and KPC-2 **d** structures shown in ribbon with position 105_β-lactamase_ shown as stick. BLIPY50 is shown as a *purple stick* in the bound form. The measurement provides the distance 105_β-lactamase_ moves upon binding to BLIP. PDB codes 1BTL and 2OV5 (apo) and 1JTG and 3E2K (bound). Images generated with Chimera

**Fig. 2:**
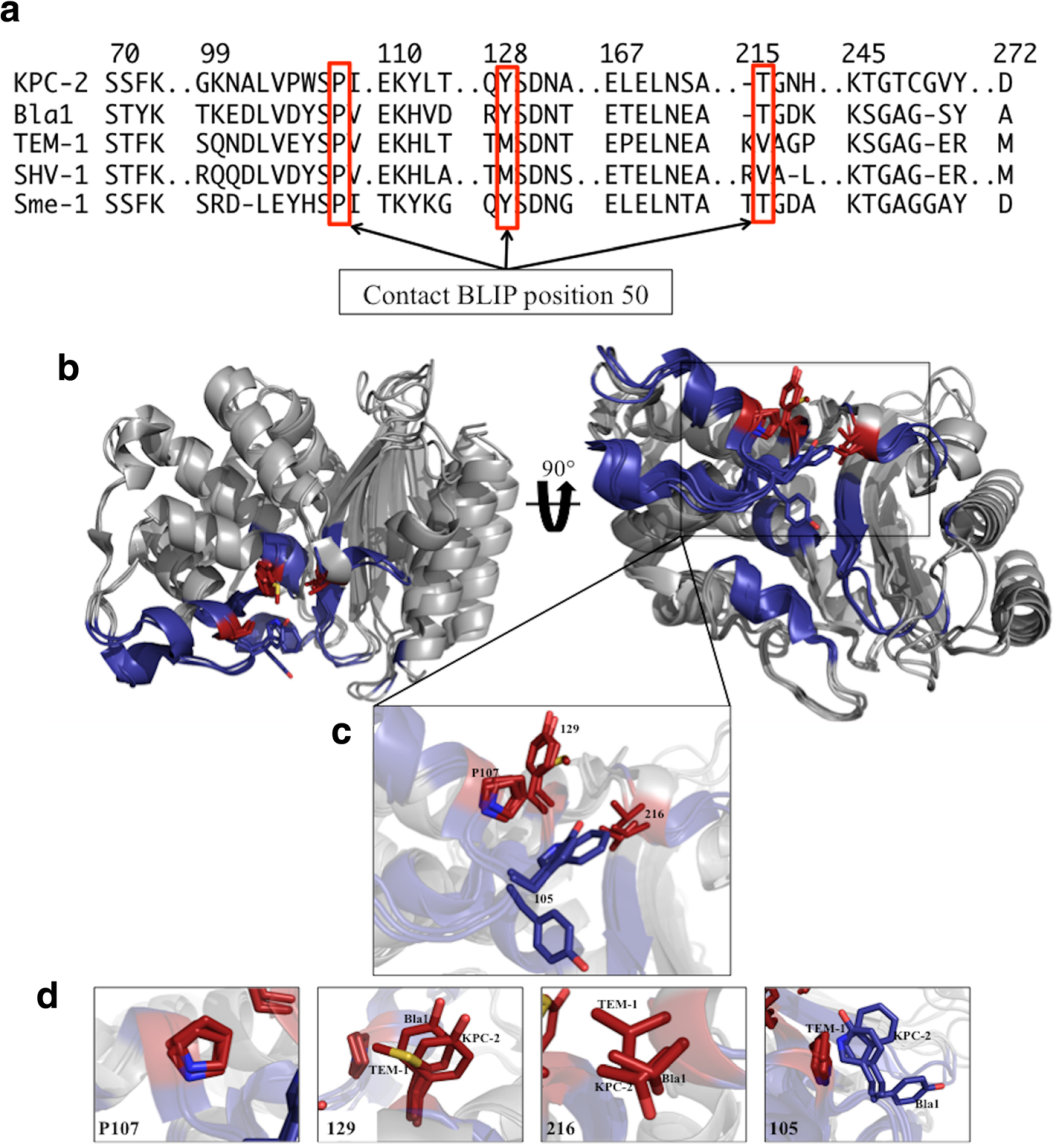
Alignment of class A β-lactamase residues at the BLIP interface. **a** The alignment is based on the structure of class A β-lactamase residues found at the BLIP interface as defined by the TEM-1/BLIP complex X-ray structure. The positions that contact BLIP position 50 are boxed in *red*. PDB codes used for the structural alignment are as follows: 2OV5 for KPC-2, 3QHY for Bla1, 1BTL for TEM-1, 1SHV for SHV-1 and 1DY6 for SME-1. Structural alignment performed in Chimera [41]. **b** β-lactamase structures are shown as *grey ribbon* and were aligned using MacPyMOL. Interface residues are shown in navy *blue* with β-lactamase position 105 shown as *blue sticks*. Residues that make direct contact with Tyr50_BLIP_ (107, 129 and 216) are shown as *red sticks*. A global structural alignment of TEM-1, Bla1 and KPC-2 β-lactamases is shown in two orientations. **c** A close-up view of an alignment of the β-lactamase residues that make contact with Tyr50_BLIP_. β-lactamase position 105 is also shown as stick model as it has been shown to make structural rearrangements upon binding to BLIP [14, 16, 35]. **d** A close up alignment of β-lactamase positions 107, 129, 216 and 105 are shown with changes in orientation made for ease of viewing the structural alignment. Residues are labeled with their corresponding β-lactamase. PDB codes used for generation of images were as follows: 1BTL for TEM-1, 3QHY for Bla1 and 2OV5 for KPC-2. Images generated in MacPyMOL

The current study focuses on further examination of the specificity determinant BLIP Tyr50 in the interaction of BLIP with various class A β-lactamases. BLIP is a 17.5 kDa protein produced from the soil bacterium *Streptomyces clavuligerus* that inhibits class A β-lactamases with varying affinities (subnanomolar to micromolar) (Table 1) [20–22]. Class A β-lactamases hydrolyze the commonly prescribed β-lactam antibiotics rendering them inactive [23]. The production of β-lactamases is the most common mechanism of bacterial resistance in Gram-negative bacteria [23]. BLIP inhibits class A β-lactamases by docking its predominantly polar, concave surface onto the enzyme, burying approximately 2,600 Å^2^ of surface area [22]. BLIP competitively inhibits class A β-lactamases via two binding loops that occlude the active site of the enzymes (Fig. 1a & b) [22]. The tertiary structures of class A β-lactamases are homologous but the sequences vary in identity from 30-70% (Fig. 2) (Table 2) [23].

**Table 2:** Sequence Identity Comparison of Class A β-lactamases

	Percent sequence identity	
	Total protein	Interface residues
KPC-2 & TEM-1	38	39
KPC-2 & Bla1	38	52
TEM-1 & Bla1	38	61

BLIP positions Tyr50, Glu73, Lys74 and Tyr143 were previously identified as specificity determinants in that substitutions at these positions result in large changes in the relative affinity of BLIP for various class A β-lactamases [13]. BLIP Tyr50 resides on the 46-53 loop that contains two hotspots for binding – Asp49 and Tyr53 [13]. A concerted rearrangement occurs at both interfaces upon binding of BLIP and TEM-1 β-lactamase; of particular interest, TEM-1 Tyr105 rearranges upon complex formation to relieve a steric clash with BLIP Tyr50 (Fig. 1c) [16, 22, 24]. In addition, residue 105, which is tryptophan in KPC-2 β-lactamase, is in a similar position as Tyr105 of TEM-1 and also undergoes a rearrangement in the BLIP-KPC-2 complex (Fig. 1d) [25]. This rearrangement of β-lactamase position 105 may be a contributing factor to changes in binding affinity upon mutation of BLIP Tyr50. BLIP Tyr50 forms van der Waals contacts with the β3 strand of TEM-1 and KPC-2, and also interacts directly with positions 107, 129 and 216 on the β-lactamase interface (Fig. 2) [16, 22]. A structural alignment of the positions on the β-lactamase interface is shown in Fig. 2 with TEM-1 and KPC-2 from the apo form and Bla1 from a BLIP-II bound form as no apo structure of Bla1 is available [26]. β-lactamase positions 107, 129 and 216 have the same sequence and similar structure for Bla1 and KPC-2 while TEM-1 differs at positions 129 and 216 (Fig. 2). As discussed above and seen in Fig. 2, the Tyr105 and Trp105 residues of TEM-1 and KPC-2 are in a similar position in apo forms of the enzyme. The Tyr105 residue of Bla1 is in an altered position in the structural alignment, however, this is likely due to the structure originating from the BLIP-II-Bla1 complex (Fig. 2) [26].

In this study, the effect of systematic substitutions at BLIP Tyr50 is examined using kinetic analysis to determine how specificity can be modulated for binding TEM-1, KPC-2 and Bla1 β-lactamases. These experiments were also performed computationally to assess the current success rate of an available protein binding prediction program. A deeper understanding of the interactions of BLIP with β-lactamases offers an opportunity to explore how specificity can be introduced into proteins rationally, by design.

## Results

### Determination of inhibition constants for β-lactamases

**Fig. 3:**
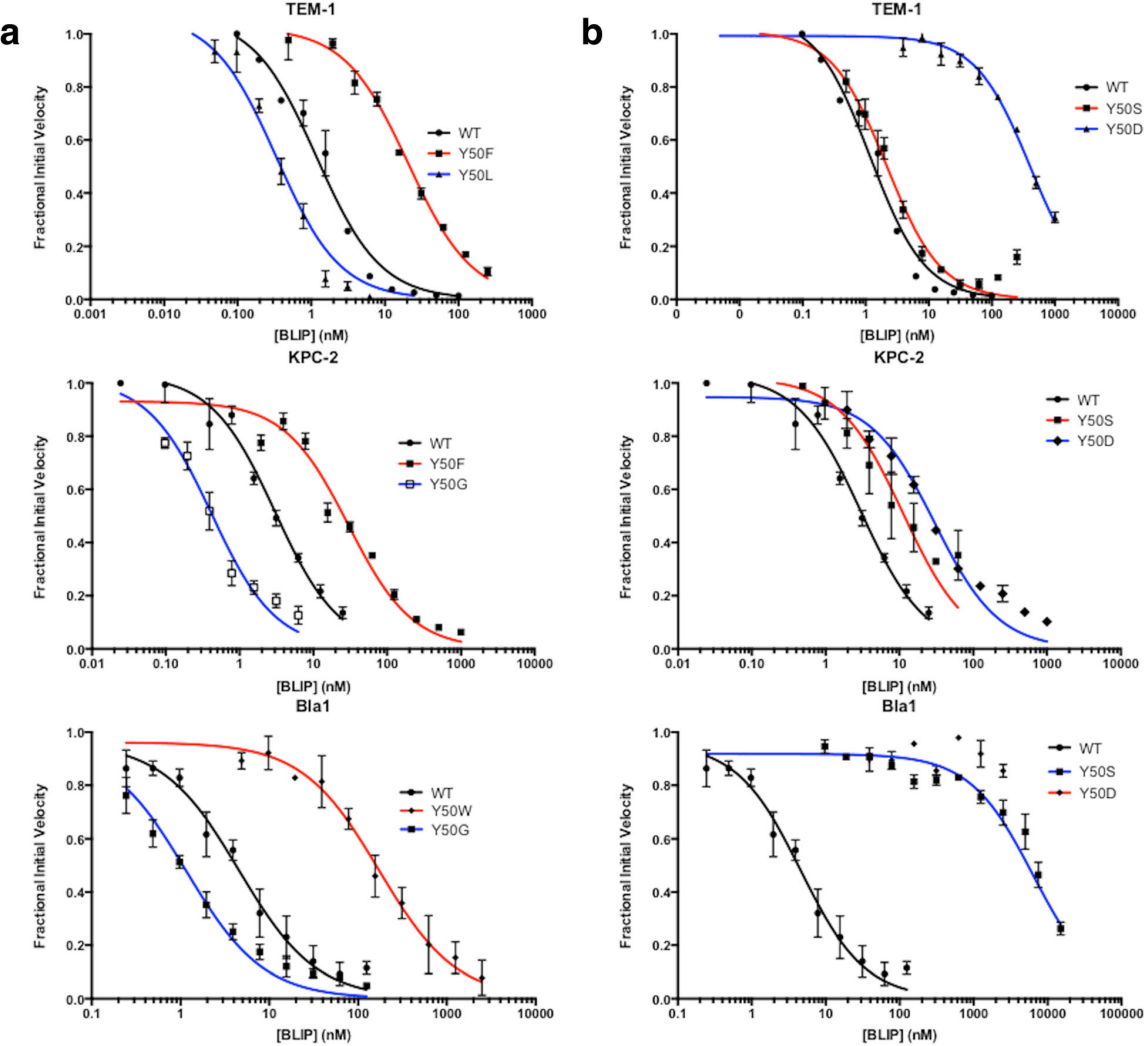
Determination of inhibition constants of BLIP mutants for binding TEM-1, KPC-2 and Bla1. The concentration of BLIP is shown on a log scale on the x-axes and fractional initial velocity is shown on the y-axes. Inhibition curves are shown for wild type BLIP in *black*. Inhibition curves for BLIP mutants that showed tighter binding than wild type are shown in *blue* while curves for mutants that showed weaker binding are shown in *red*

BLIP Y50 was substituted to all 19 amino acids to investigate the role of this residue in modulating specificity. The mutant proteins were purified (with the exception of BLIP Y50I which could not be purified due to low expression and yield) and assayed with TEM-1, KPC-2 and Bla1 β-lactamases to determine the inhibition constants (Table 3, Fig. 3). It was previously reported that the BLIP Y50A substitution alters the binding specificity for β-lactamases; however, the extent to which other substitutions at position 50 alter binding specificity is unknown. Overall, the results of this study support the hypothesis that BLIP Y50 makes important contributions to the binding specificity of BLIP for class A β-lactamases.

**Table 3:** Inhibition Constants of BLIPY50 mutants for Class A β-lactamases

	BLIP Mutant	Ki (nM) KPC-2	Ki (nM) TEM-1	Ki (nM) Bla1
Nonpolar	WT	1.5 ± 0.2	0.5 ± 0.1	2.5 ± 0.3
	Y50A	1.9 ± 0.3	0.010 ± 0.001	1.2 ± 0.3
	Y50F	15 ± 2	10.3 ± 0.8	90 ± 14
	Y50L	0.7 ± 0.2	0.17 ± 0.02	160 ± 39
	Y50M	0.5 ± 0.1	0.11 ± 0.01	34 ± 9
	Y50V	10 ± 1	3.5 ± 0.6	200 ± 11
	Y50W	1.1 ± 0.2	11 ± 1	90 ± 11
Polar	Y50C	42 ± 6	60 ± 10	360 ± 5
	Y50N	3.8 ± 0.8	7.8 ± 0.5	180 ± 43
	Y50Q	1.0 ± 0.5	0.24 ± 0.02	1.3 ± 0.3
	Y50S	5 ± 1	1.1 ± 0.1	3110 ± 5
	Y50T	3.5 ± 0.4	2.5 ± 0.2	290 ± 53
Small	Y50G	0.72 ± 0.02	0.40 ± 0.06	0.58 ± 0.07
	Y50P	5 ± 1	40 ± 3	220 ± 28
Charged	Y50H	1.4 ± 0.1	5.0 ± 0.1	230 ± 2
	Y50R	11 ± 1	69 ± 2	>1500
	Y50K	260 ± 10	195.3 ± 0.2	180 ± 47
	Y50D	3.2 ± 0.3	160 ± 23	>4500
	Y50E	5.5 ± 0.1	76 ± 6	331 ± 6

BLIP Y50I could not be purified

**Fig. 4:**
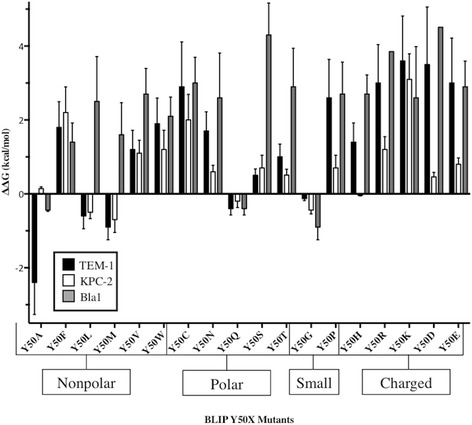
Comparison of ΔΔG values of BLIP mutants for binding TEM-1, KPC-2 and Bla1 β-lactamases. BLIP Y50X mutants are shown on the x-axis and the calculated change in free energy is shown on the y-axis. Values for TEM-1 are shown in *black*, KPC-2 in *white* and Bla1 in *gray*

BLIP is a potent inhibitor of each of the β-lactamases studied with K_i_ values of 0.5 nM for TEM-1, 1.5 nM for KPC-2 and 2.5 nM for Bla2 (Table 3). The effect of substitutions at BLIP Y50 on the binding affinity for the enzymes, however, is widely different. Most BLIP Y50 substitutions retain tight binding for KPC-2 while many substitutions reduce binding to TEM-1 and the majority of substitutions are detrimental for binding Bla1 (Table 3). This is apparent from the finding that only 3 substitutions result in a greater than 10-fold loss in affinity for KPC-2 while 10 substitutions reduce binding by >10-fold for TEM-1 and 15 result in a >10-fold loss in affinity for Bla1 (Table 3). The changes in binding constants for the substitutions were normalized by calculating the changes in free energy of the complex using the following equation: ΔΔG = -RT ln (K_i_^WT^ / K_i_^MUT^) (Table 4). A negative ΔΔG value is indicative of an increase in binding affinity as compared to wild type while a positive value corresponds to a decrease in binding affinity. Using these values, the wide difference in tolerance to BLIP Y50 substitutions for binding β-lactamases is clear in that the average effect of substitutions (∆∆G) on binding KPC-2 was 0.7 kcal/mol while that for binding TEM-1 was 1.3 kcal/mol and that for binding Bla1 was 2.3 kcal/mol (Table 4 and Fig. 4). These results indicate that the sequence requirements at BLIP position 50 are significantly less stringent for binding KPC-2 compared to the requirements for binding TEM-1 and Bla1.

**Table 4:** ΔΔG values for Class A β-lactamases and BLIP Y50 mutants

	BLIP Mutant	ΔΔG(kcal/mol) KPC-2	ΔΔG(kcal/mol) TEM-1	ΔΔG(kcal/mol) Bla1
Nonpolar	Y50A	0.14 ± 0.03	-2.4 ± 0.5	-0.44 ± 0.07
	Y50F	2.2 ± 0.4	1.8 ± 0.4	1.4 ± 0.3
	Y50L	-0.5 ± 0.1	-0.6 ± 0.2	2.5 ± 0.7
	Y50M	-0.7 ± 0.2	-0.9 ± 0.2	1.6 ± 0.5
	Y50V	1.1 ± 0.2	1.2 ± 0.3	2.7 ± 0.4
	Y50W	1.2 ± 0.3	1.9 ± 0.4	2.1 ± 0.4
Polar	Y50C	2.0 ± 0.4	2.9 ± 0.7	3.0 ± 0.4
	Y50N	0.6 ± 0.1	1.7 ± 0.3	2.6 ± 0.7
	Y50Q	-0.2 ± 0.1	-0.4 ± 0.1	-0.4 ± 0.1
	Y50S	0.7 ± 0.2	0.5 ± 0.1	4.3 ± 0.5
	Y50T	0.51 ± 0.09	1.0 ± 0.2	2.9 ± 0.6
Small	Y50G	-0.44 ± 0.06	-0.13 ± 0.03	-0.9 ± 0.2
	Y50P	0.7 ± 0.2	2.6 ± 0.6	2.7 ± 0.5
Charged	Y50H	0.042 ± 0.006	1.4 ± 0.3	2.7 ± 0.3
	Y50R	1.2 ± 0.2	3.0 ± 0.6	>3.9
	Y50K	3.1 ± 0.4	3.6 ± 0.7	2.6 ± 0.7
	Y50D	0.46 ± 0.07	3.5 ± 0.9	>4.5
	Y50E	0.8 ± 0.1	3.0 ± 0.7	2.9 ± 0.4

BLIP Y50I could not be purified

Examination of the substitution results in Tables 3 and 4 reveals some common sequence requirements at BLIP position 50 for binding all three β-lactamases. For example, cysteine, phenylalanine and lysine substitutions showed a greater than 10-fold decrease in binding affinity for all three enzymes (Table 3). Cysteine may decrease binding affinity because of the potential of being oxidized, which would disrupt binding. Phenylalanine has a similar van der Waals volume as the wild-type tyrosine residue but does not have the hydrogen bonding capacity and could potentially interrupt the organization of structural waters at the interface because of its strong hydrophobic properties. The decrease in binding affinity when lysine is substituted at BLIP position 50 is likely due to introduction of an unpaired charge in the interface. Although lysine was the only charged residue to globally decrease binding affinity by 10-fold, the general finding is that charged residues at position 50 result in a decrease in binding affinity for all β-lactamases tested, although the effect is less pronounced for binding KPC-2 (Table 3). This is supported by the fact that BLIP containing arginine, glutamate or aspartate at position 50 exhibited decreased affinity for TEM-1 and Bla1 by greater than 100-fold and also exhibited decreased affinity for KPC-2 (Table 3). A proline at position 50 is also generally disruptive in that it decreased the binding affinity of BLIP for all three β-lactamases, possibly by altering the conformation or flexibility of the Y50 loop, which includes two hot spot residues for binding [27].

Another common trend for binding all three β-lactamases is that substitutions of BLIP Y50 by the small amino acids alanine and glycine either does not affect or improves affinity (Table 3). For example, BLIP Y50A retains affinity for KPC-2 and Bla1 and exhibits 50-fold tighter binding of TEM-1 while Y50G shows a small increase in affinity for all three enzymes. As noted above and shown in Figs. 1 and 2, Tyr105 in TEM-1 and the equivalent Trp105 in KPC-2 change position in the BLIP-β-lactamase complexes compared to the apo-enzymes in order to avoid a steric clash with BLIP Y50. It is possible that substitution of Y50 with alanine or glycine avoids the clash and allows β-lactamase residue 105 to retain its apo-position in the complex, which may result in improved affinity.

Finally, polar residue substitutions at BLIP Y50 have quite disparate effects on binding the β-lactamases. For example, serine and threonine substitutions have relatively small effects on binding KPC-2 and TEM-1 but result in greatly decreased binding to Bla1 (Table 3). In addition, glutamine at BLIP position 50 does not affect binding to any of the β-lactamases while an asparagine substitution results in decreased affinity for all three β-lactamases, including a greater than 10-fold decrease for binding TEM-1 and Bla1 (Table 3). This result cannot easily be explained because asparagine has similar properties to glutamine, which had little to no effect on binding any of the β-lactamases.

### Impact of BLIP Y50 on binding specificity

Because the purpose of this study was to examine the role of BLIP position 50 as a specificity determinant, substitutions that have differential effects on binding are of interest. As indicated above and is apparent in Tables 3 and 4, many substitutions at BLIP Y50 have differential effects on β-lactamase binding, the most clear example being the numerous substitutions that retain or modestly impact binding to KPC-2 while greatly decreasing binding to Bla1 (Y50-L,M,W,N,S,T,H), and the subset of substitutions that retain binding to KPC-1 and TEM-1 while losing affinity for Bla1 (Y50-L,M,S,T). In contrast, there are no BLIP Y50 substitutions that retain affinity for Bla1 while losing affinity for KPC-2 or TEM-1 (Table 3). Thus, the large differences in stringency of sequence requirements at position 50 results in BLIP variants that bind KPC-2 but not TEM-1 and Bla1 as well as those that bind KPC-2 and TEM-1 but not Bla1. However, substitutions at BLIP Y50 do not produce a variant that binds Bla1 but not TEM-1 or KPC-2.

It was next of interest to examine a possible structural basis for the observed differences in sequence requirements for BLIP Y50 substitutions for binding Bla1 versus KPC-2 and TEM-1. The side chain of BLIP Y50 is in direct contact with β-lactamase residues 107, 129 and 216 in the crystal structures of the BLIP-TEM-1 and BLIP-KPC-2 complexes (Fig. 2). These β-lactamase contact residues are identical between KPC-2 and Bla1 (P107-Y129-T216) while TEM-1 differs at 2 of the 3 positions (P107-M129-V216) (Fig. 2). Based on these sequences, it would be expected that substitutions at BLIP Y50 would have similar effects on binding KPC-2 and Bla1. The results indicate this is clearly not the case. Therefore, a simple comparison of the β-lactamase contact residues for BLIP Y50 does not explain the observed differences in effects of substitutions on binding the β-lactamases. Although KPC-2 and Bla1 have the same amino acids at positions 107, 129 and 216, the overall sequence identity of all β-lactamase residues at the interface is higher between Bla1 and TEM-1 compared to KPC-2 (Table 2). There are a total of 10 positions (71, 102, 106, 109, 112, 133, 172, 246, 248 and 249) on the β-lactamase interface where TEM-1 and Bla1 have the same sequence and the sequence of KPC-2 differs (Fig. 2). KPC-2 is much better at accommodating changes at BLIP Y50 than both Bla1 and TEM-1 and has the most sequence differences at the interface. Therefore, more widespread differences in the entire interface may influence the effect of substitutions at BLIP Y50. This may be due to changes at the interface induced by mutation of BLIP Y50 that propagate outside of its local environment. In fact, previous studies have shown that BLIP Tyr50 is energetically coupled to both positions Tyr143 and Glu73, which are not in direct contact with Tyr50 [13]. The hypothesis that changes at position 50 are influenced by other sites in the interface and vice versa is consistent with previous observations of structural plasticity and cooperativity of the BLIP interface upon mutation [12, 13, 15, 16, 27]. For example, it has been shown that the BLIP W150A mutation induces a greater than 4 Å shift in residue Asp49, demonstrating both structural flexibility of the loop containing Tyr50 and long distance coupling at the BLIP interface [28].

An interesting question is whether there are also more stringent sequence requirements at other BLIP positions in the interface for binding Bla1 versus TEM-1 and KPC-2. A previous alanine-scanning mutagenesis study for 23 BLIP residues that contact β-lactamase in the bound complex evaluated binding to TEM-1 and Bla1 (KPC-2 was not evaluated) [27]. The results of this study suggest that the sequence requirements are not generally more stringent for BLIP binding Bla1 in that the average ΔΔG effect for the 23 alanine substitutions was less detrimental for binding Bla1 (avg ΔΔG = 0.4) than for TEM-1 (avg ΔΔG = 1.0) [27]. Therefore, the stringent sequence requirements observed here for Bla1 binding are unique to position 50 and not a general property of all interface positions for binding Bla1.

### Comparison of experimental and predicted ΔΔG values

It is appealing to use computational methods to guide engineering of binding specificity in protein-protein interactions. Therefore, we were interested to examine how computational methods compared to our experimental results. The same mutagenesis experiment was performed computationally on BLIP position 50 with the BeAtMuSiC server, which computes theoretical ΔΔG values based on a set of statistical potentials derived from known protein structures [11]. The TEM-1-BLIP (PDB code: 1JTG) and KPC-2-BLIP (PDB code: 2OV5) complexes were submitted for analysis. The BLIP-Bla1 interaction was not analyzed because there is currently no crystal structure available of this complex.

**Fig. 5:**
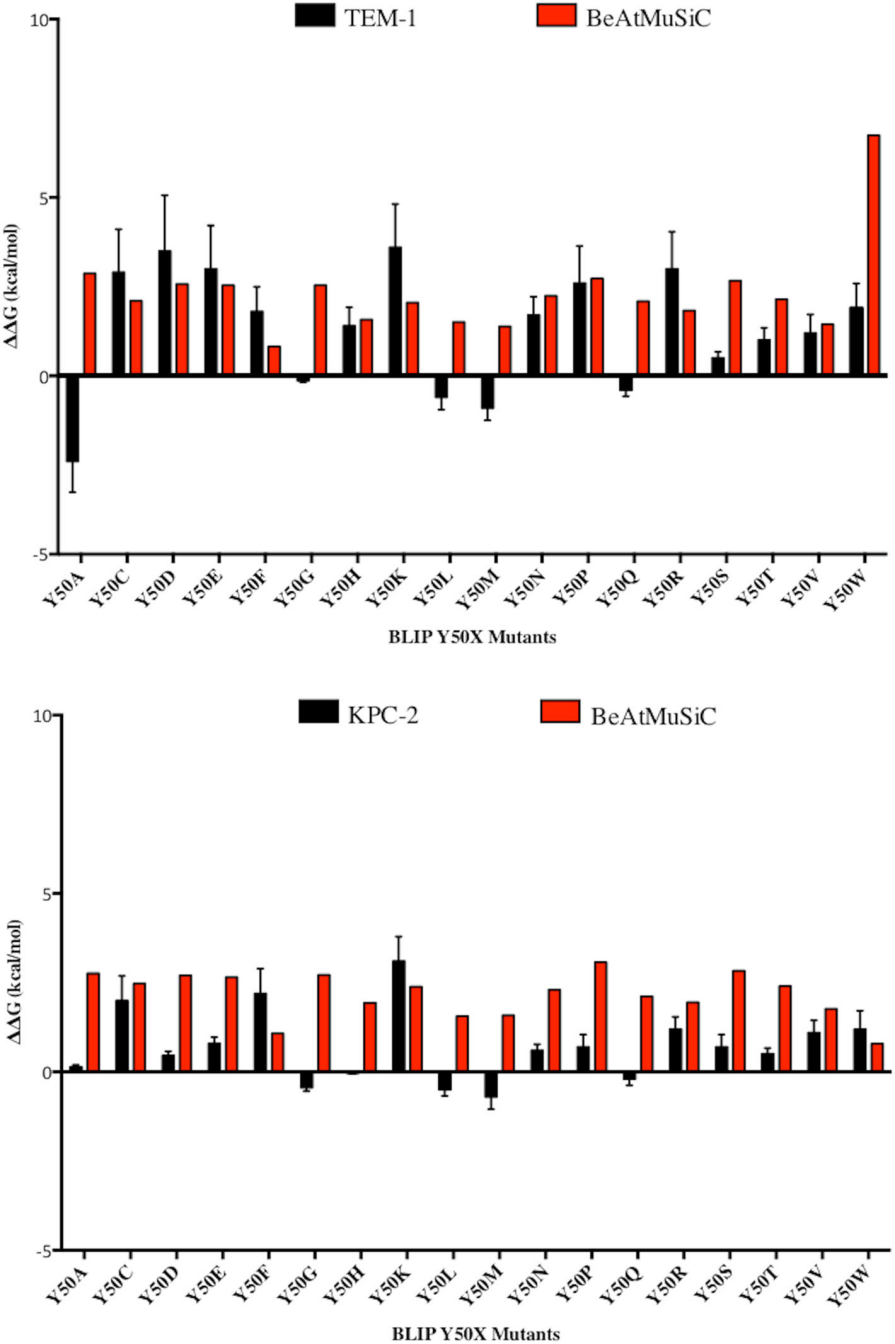
Comparison of experimental and predicted ΔΔG values for BLIP Y50 variants. A comparison of the experimental and predicted ΔΔG values of the BLIP Y50X mutants for binding TEM-1 are shown on the top panel. A comparison of the experimental and predicted ΔΔG values of the BLIP Y50X mutants for binding KPC-2 are shown on the bottom panel. The BeAtMuSiC server was used to generate predicted ΔΔG values. The predicted values are shown in *red* and the experimental values are shown in *black*

The ΔΔG values generated by the BeAtMuSiC server and the experimentally determined ΔΔG values are plotted in Fig. 5. The average predicted ΔΔG value for the TEM-1/BLIP interaction was 2.0 kcal/mol while the experimentally determined average ΔΔG value was 1.3 kcal/mol. For the KPC-2-BLIP interaction, the average predicted ΔΔG was 2.2 kcal/mol and the experimentally determined average ΔΔG was 0.7 kcal/mol. Therefore, the BeAtMuSiC server was more accurate at predicting ΔΔG values for the BLIP-TEM-1 interaction than the BLIP-KPC-2 interaction.

## Discussion

Specificity determinants are often identified through alanine scanning of interface residues [16–18, 29, 30]. Whether mutations to other amino acids would also identify these residues as specificity determinants is unknown. Here, we present data supporting the role of BLIP Y50 as a specificity determinant and furthermore, provide evidence that this position can be targeted to engineer binding specificity of BLIP for a range of class A β-lactamases.

The BeAtMuSiC server did not predict any negative changes in free energy meaning that the energy of the wild-type complex is predicted to be more stable than any of the mutants. However, the BLIP Y50A substitution was shown experimentally to bind TEM-1 β-lactamase 50-fold tighter than wild type BLIP. It is known that some residues rearrange upon binding of BLIP and class A β-lactamases and this could influence the accuracy of the prediction programs [27]. These rearrangements could play into the differences seen (about 1.4 kcal/mol) between our experimental ΔΔG values and the predicted values. Furthermore, BLIP Y50 is located on a loop that has two hot spots for binding; therefore, even small alterations in the placement of this loop induced by Y50 substitutions could result in large changes in binding affinity. In addition, as described above, the effect of substitutions at BLIP Y50 may be influenced by positions outside of the direct contact residues through coupled interactions and therefore predictions of effects of substitutions poses a significant challenge for computational prediction programs. However, it may be these same properties that provide BLIP with its unique ability to bind structurally homologous proteins with a wide-range of affinities.

Because BLIP binds homologous β-lactamase structures that only differ by small changes in sequence at the interface, the BLIP-β-lactamase system is useful for examining how sequence dictates binding affinity. However, an alignment of the β-lactamase sequences in direct contact with the BLIP Y50 residue (TEM-1 residues 107, 129 and 216) suggests that KPC-2 and Bla1 would exhibit the same changes in binding affinity upon mutation of BLIP Y50 because they have the same amino acids in similar conformations at these positions; however, this was not the case (Fig. 2). Although KPC-2 and Bla1 have the same amino acids at positions 107, 129 and 216 that directly interact with Y50, the overall sequence identity of all β-lactamase residues at the interface is higher between Bla1 and TEM-1 compared to KPC-2 (Table 2). KPC-2 was much better at accommodating changes at BLIP Y50 than both Bla1 and TEM-1 and had the most sequence differences at the interface. This suggests that simply comparing sequence identity of positions that make direct interactions with BLIP Y50 (or any BLIP residue) is not sufficient to predict changes in binding affinity upon mutation. Therefore, more widespread differences in the entire interface may influence the effect of substitutions at BLIP Y50. This may be due to changes at the interface induced by mutation of BLIP Y50 that propagate outside of its local environment due to structural plasticity and coupled interactions.

## Conclusions

Properties such as structural plasticity and cooperativity between residues are important for mediating protein interactions and critical for allosteric regulation in various cell processes [31–34]. Understanding how these properties contribute to binding specificity would greatly improve current protein binding prediction programs. This is an active area of investigation in G-protein coupled receptors, the human growth hormone receptor and other proteins [31–34]. Numerous studies such as these have established that the dynamic nature of proteins is critical to binding and proper functioning; however, this dynamic nature is challenging to predict and structurally understand, as flexible proteins are inherently difficult to model and crystallize. Here, we demonstrate the complexity of predicting the impact of substitutions using the well-studied BLIP-β-lactamase protein-protein interaction model. Furthermore, we have shown that surveying sequence homology and the structural interface of a complex are not sufficient in predicting the impact of mutations.

Currently, protein prediction programs are unable to reliably predict changes in binding affinity upon mutation at the protein interface. Systematic studies such as these could improve the current state by providing experimental data to be incorporated into these programs. Lastly, there is a pressing need for new detection methods for β-lactamases, which are a widespread source of resistance to β-lactam antibiotics. Identification of specificity determinants in BLIP could be useful in the development of BLIP-based diagnostic reagents that can discriminate between class A β-lactamases and inform treatment options for clinicians.

## Methods

### Construction of BLIP Y50 mutants

BLIP position 50 was mutated to all 19 amino acids using the Quickchange method (Stratagene) and *Pfu* polymerase (Stratagene) on the pGR32 plasmid with an N-terminal His-tag as previously described [35]. DNA sequencing was used to confirm the mutations and that no extraneous mutations occurred elsewhere on the BLIP gene in each of the mutants (Lonestar Labs).

### Protein purification

N-terminal His-tagged BLIP mutants were purified using the TALON Metal Affinity Resin (Clontech) [35]. Despite multiple attempts, the BLIP Y50I mutant could not be purified due to poor expression and yield. The TEM-1 and Bla1 proteins were purified as previously described using a zinc chelating column and elution with a pH gradient [36]. KPC-2 was purified as previously described using a HiTrap SP column and elution with an NaCl gradient [37]. The BLIP mutants and the various β-lactamases were each concentrated and injected onto a Superdex 75 gel filtration size exclusion column as a final purification step. Fractions with greater than 90% purity as determined by SDS-PAGE were combined, concentrated and used in the inhibition assay. The protein concentrations for the β-lactamases and BLIP mutants were determined by a Bradford assay where they were compared with a curve that was calibrated by quantitative amino acid analysis specific to each protein. The concentrations for all proteins were confirmed by measuring absorbance at 280 nm and using the extinction coefficient as determined by the ExPASy ProtParam tool [38]. Kinetic parameters (*k*_*cat*_ and K_m_) were determined to confirm activity for each β-lactamase using the chromogenic β-lactam substrate, nitrocefin (data not shown).

### β-lactamase inhibition assay

Inhibition constants for BLIP mutants binding to the β-lactamases were determined as previously described [35]. Increasing concentrations of BLIP were incubated with a constant concentration of β-lactamase (1 nM) for 1 h at room temperature in 50 mM sodium phosphate buffer pH 7.0. The chromogenic substrate, nitrocefin, was then added at the K_m_ concentration for the β-lactamases and the initial velocity was measured at 482 nm in 20 s intervals. The experiments were performed in at least duplicate. The K_i_^app^ for each BLIP mutant was determined by fitting the initial velocities to the Morrison tight-binding equation [39]:

1 $$ {E}_{\mathrm{free}}=\left[{E}_0\right]-\frac{\left[{E}_0\right]+\left[{I}_0\right]+{\displaystyle {K}_i^{\mathrm{app}}}-\sqrt{{\left(\left[{E}_0\right]+\left[{I}_0\right]+{\displaystyle {K}_i^{\mathrm{app}}}\right)}^2-\left(4\left[{E}_0\right]\left[{I}_0\right]\right)}}{2} $$

where E_free_ is the concentration of free enzyme determined by residual activity of the β-lactamase by comparison with the initial velocity of nitrocefin hydrolysis by the uninhibited β-lactamase, [E_0_] is the total enzyme concentration and [I_0_] is the total BLIP concentration. The errors reported were calculated based on the fit of the curve. The K_i_ values were calculated from the K_i_^app^ values as previously described using eq. 2 [40]:

2 $$ \mathrm{K}\mathrm{i} = {{\mathrm{K}}_{\mathrm{i}}}^{\mathrm{app}}/\left(1+\left(\left[\mathrm{S}\right]/{\mathrm{K}}_{\mathrm{M}}\right)\right) $$

### ΔΔG calculations

ΔΔG was calculated using the following equation:

3 $$ \varDelta \varDelta \mathrm{G} = - \mathrm{R}\mathrm{T}\  \ln\ \left({\displaystyle {\mathrm{K}}_{\mathrm{i}}^{\mathrm{WT}}}/{\displaystyle {\mathrm{K}}_{\mathrm{i}}^{\mathrm{MUT}}}\right) $$

Using this equation, a decrease in K_i_ upon mutation would result in a negative ΔΔG value while an increase in K_i_ would be reported as a positive change in free energy. Error for ΔΔG values was calculated using the following equation:

4 $$ \varDelta \varDelta \mathrm{G}\ \mathrm{error} = \varDelta \varDelta \mathrm{G}\sqrt{\frac{{\mathrm{SEM}}^2}{{\displaystyle {\mathrm{K}}_{\mathrm{i}}^{\mathrm{WT}}}}+\frac{{\mathrm{SEM}}^2}{{\displaystyle {\mathrm{K}}_{\mathrm{i}}^{\mathrm{MUT}}}}} $$

Where ‘SEM’ represents standard error of the mean, ‘WT’ represents wild-type BLIP and ‘MUT’ represents the mutant protein.

### Computational prediction of the effect of BLIP mutations on binding affinity

The BeAtMuSiC online server was used to predict changes in binding affinity of the BLIP mutants on complex formation with TEM-1 and KPC-2 β-lactamases [11]. The BeAtMuSiC server relies on a set of statistical potentials derived from known protein structures and predicts the changes in binding affinity by the combined effect of the mutation on the overall stability of the complex and the interface [11]. PDB codes 1JTG (BLIP/TEM-1) and 3E2K (BLIP/KPC-2) (chains A and B) were submitted for analysis.

## Abbreviation

BLIP: β-lactamase inhibitory protein
